# Targeted Analysis of Serum and Urinary Metabolites for Early Chronic Kidney Disease

**DOI:** 10.3390/ijms26072862

**Published:** 2025-03-21

**Authors:** Mihaela-Roxana Glavan, Carmen Socaciu, Andreea Iulia Socaciu, Oana Milas, Florica Gadalean, Octavian M. Cretu, Adrian Vlad, Danina M. Muntean, Flaviu Bob, Anca Suteanu, Dragos Catalin Jianu, Maria Stefan, Lavinia Marcu, Silvia Ienciu, Ligia Petrica

**Affiliations:** 1 Department of Internal Medicine II—Nephrology, “Victor Babeș” University of Medicine and Pharmacy, Eftimie Murgu Sq. No. 2, 300041 Timișoara, Romania; patruica.mihaela@umft.ro (M.-R.G.); gadalean.florica@umft.ro (F.G.); bob.flaviu@umft.ro (F.B.); anca.simulescu@umft.ro (A.S.); maria.mogos@umft.ro (M.S.); lavinia.balint@umft.ro (L.M.); silvia-ioana.ienciu@umft.ro (S.I.); petrica.ligia@umft.ro (L.P.); 2 Centre for Molecular Research in Nephrology and Vascular Disease, Faculty of Medicine, “Victor Babeș” University of Medicine and Pharmacy, 300041 Timișoara, Romania; 3 Research Center for Applied Biotechnology and Molecular Therapy BIODIATECH, SC Proplanta, Str. Trifoiului 12G, 400478 Cluj-Napoca, Romania; csocaciudac@gmail.com; 4 Department of Occupational Health, University of Medicine and Pharmacy “Iuliu Haţieganu”, Str. Victor Babes 8, 400347 Cluj-Napoca, Romania; andreeaiso@gmail.com; 5 Department of Surgery—Surgical Semiotics, “Victor Babeş” University of Medicine and Pharmacy, Eftimie Murgu Sq. No. 2, 300041 Timişoara, Romania; octavian.cretu@umft.ro; 6 Department of Internal Medicine II—Diabetes and Metabolic Diseases, “Victor Babeș” University of Medicine and Pharmacy, Eftimie Murgu Sq. No. 2, 300041 Timișoara, Romania; vlad.adrian@umft.ro; 7 Department of Functional Sciences—Pathophysiology, Faculty of Medicine, “Victor Babeș” University of Medicine and Pharmacy, Eftimie Murgu Sq. No. 2, 300041 Timișoara, Romania; daninamuntean@umft.ro; 8 Center for Translational Research and Systems Medicine, Faculty of Medicine, “Victor Babeș” University of Medicine and Pharmacy, Eftimie Murgu Sq. No. 2, 300041 Timișoara, Romania; 9 Department of Neurosciences—Neurology, “Victor Babeș” University of Medicine and Pharmacy, Eftimie Murgu Sq. No. 2, 300041 Timișoara, Romania; jianu.dragos@umft.ro; 10 Centre for Cognitive Research in Neuropsychiatric Pathology, Clinical County Emergency Hospital, “Victor Babes” University of Medicine and Pharmacy, Liviu Rebreanu Ave. No 156, 300041 Timișoara, Romania

**Keywords:** chronic kidney disease, metabolomics, biomarkers, amino acids, uremic toxins, acylcarnitines

## Abstract

Chronic kidney disease (CKD) has become one of the most rapidly advancing diseases of the century, contributing significantly to increased mortality and morbidity. Metabolomics presents a promising approach to understanding CKD pathogenesis and identifying novel biomarkers for early diagnosis. This study evaluated serum and urine metabolomic profiles in CKD patients with declining glomerular filtration rates (eGFR). Using targeted metabolomics, we quantified seven potential metabolites in blood and urine samples from 20 healthy individuals and 99 CKD patients staged by eGFR according to the KDIGO guidelines. The analysis was conducted using ultra-high performance liquid chromatography combined with electrospray ionization quadrupole time-of-flight mass spectrometry. The metabolites investigated included L-phenylalanine, L-methionine, arginine, indoxyl sulfate, kynurenic acid, and L-acetylcarnitine. Quantitative assessments were performed using pure standards and validated through methods such as the limit of detection (LOD) and limit of quantification (LOQ). The findings identified potential biomarkers for early CKD diagnosis: in serum, L-phenylalanine, L-methionine, arginine, kynurenic acid, and indoxyl sulfate, while L-acetylcarnitine was significant in urine. These biomarkers could provide valuable insights into CKD progression and support in developing more effective diagnostic tools for early intervention.

## 1. Introduction

The kidney plays an essential role in maintaining homeostasis in the human body by heaving several vital functions, such as hormone secretion, acid–base balance, hydroelectrolitic balance, and regulating plasma volume. A decrease under 60 mL/min/1.73 m^2^ in the glomerular filtration rate (GFR) for at least 3 months defines the pathology known as chronic kidney disease (CKD), irrespective of the cause. Many pathologies can alter kidney functions, resulting in CKD [1]. The resulting disruption of blood filtration leads to a chronic and refractory form of CKD, which impacts quality of life and increases mortality risk regardless of its underlying cause. The incidence of CKD has risen significantly, making it a leading cause of mortality worldwide [2].

Several epidemiological studies and systemic, comprehensive reviews suggest that the worldwide prevalence of CKD has surpassed that of diabetes mellitus, impacting an estimated 11–13% of the global population [2]. CKD is characterized by accelerated deterioration of renal function, potentially culminating in a spectrum of complications and ultimately progressing to end-stage renal disease (ESRD) [3].

Despite the increasing prevalence of renal pathologies, the current diagnostic methods for CKD are limited, often leading to a diagnosis in the advanced stages of the disease. As CKD progresses to ESRD, several metabolites and uremic solutes accumulate in the plasma. This observation underscores the urgent need for novel biomarkers to enable early diagnosis, track disease progression, and identify potential therapeutic targets in nephrology [4].

The complex interconnections between circulating metabolites’ levels and the kidney are well known but insufficiently explored. The impact of the kidneys on the metabolic pathways and circulating metabolite levels are complex and heterogeneous [5]. The kidneys are crucial for excreting metabolic waste and reabsorbing essential nutrients. Proximal tubule epithelial cells are responsible for producing several amino acids, and the metabolite production rate can be affected by gut microbiome dysbiosis and heightened insulin resistance. As renal function declines, the excretion of metabolic byproducts increases, resulting in elevated circulating levels of detrimental metabolites [6].

Metabolomics, a powerful tool for assessing metabolic pathways, has provided innovative insights into physiopathology, the mechanisms, and the progression of CKD [6]. Recent research has indicated that metabolites, particularly those related to the amino acid class, could significantly influence the onset and progression of chronic diseases, including CKD [7]. These data underscore the potential of metabolomics in revolutionizing our understanding and management of CKD.

Arginine, classified as a semi-essential amino acid and its analogs, has been implicated in numerous chronic diseases, highlighting its significant biological relevance. The end products of arginine metabolism and arginine itself have been associated with immune responses, the modulation of inflammation, and fibrosis [7].

Several studies have indicated an association between diminished serum arginine levels and CKD. As a result, arginine and its metabolic pathways emerge as prospective therapeutic targets in the strategic management of CKD [8]. However, it is essential to note that our understanding of arginine’s role in CKD is still evolving, and more research is needed to clarify its functions and potential therapeutic applications.

L-methionine (Met), an essential amino acid involved in numerous metabolic pathways, has been associated with an augmentation of oxidative stress [9] and subsequent infliction of vascular and renal damage, including tubular hypertrophy [10]. Furthermore, it appears that the kidneys significantly contribute to Met metabolism, thereby establishing a connection between methionine and the onset and progression of renal pathology [11]. A study on murine subjects on a diet with a deficiency in Met and choline pointed out changes in several metabolic pathways, including carnitine, arginine, serine, and choline [10]. Previous studies have shown that Met levels were correlated with a reduction in kidney function [12,13].

Phenylalanine is another essential amino acid that plays a vital role in the function and structure of proteins and enzymes and the biosynthesis of other amino acids. In CKD, the metabolic conversion of phenylalanine to tyrosine is impaired, leading to decreased plasma and tissue concentrations of tyrosine, phenylalanine, and the ratio of tyrosine to phenylalanine [14]. Additionally, these interconnected amino acids are linked to an increased incidence of diabetic retinopathy (DR) in individuals with type 2 diabetes mellitus [15]. Therefore, phenylalanine could be used to predict CKD prevalence and diagnosis.

Uremic toxins accumulate within the human organism as renal functionality deteriorates. Indoxyl sulfate (IS), classified as a uremic toxin, is associated with the development and progression of CKD. Kynurenic acid (KYNA), a kynurenine pathway metabolite, represents the primary degradation route for tryptophan and modulates immune responses, inflammation, and energy metabolism [16]. The kidneys are the primary organs for synthesizing, eliminating, and degrading tryptophan end products [17]. In patients with kidney dysfunction, the kynurenine pathway is disturbed. Recent studies indicate that metabolites from the kynurenine pathway, including KYNA, undergo alterations in CKD. This alteration has also been associated with the progression of CKD, uremia, and vascular complications [18]. Hence, the exact role of KYNA and its implications in CKD are complex and incriminate multiple metabolic pathways.

Carnitines, a distinct category of molecules, are implicated in the delivery of lipids to mitochondria, the metabolism of amino acids, the modulation of inflammatory responses, and the mitochondria activity [19]. A study conducted by Xia et al. discovered a correlation between elevated acylcarnitine levels and poor prognosis and treatment response in IgA nephropathy [20]. Furthermore, a study conducted by Yano et al. identified elevated serum concentrations of acylcarnitine in patients with CKD [21].

The current study follows a prior untargeted metabolomic analysis of serum and urinary metabolites in CKD patients. Its objective is to conduct a targeted analysis of six metabolites that were identified as potential biomarkers for CKD.

## 2. Results

### 2.1. Untargeted Multivariate and Univariate Analyses

In a prior study of the same cohort of subjects, untargeted multivariate and univariate statistics were conducted after the UHPLC-QTOF-ESI ± MS analysis [22]. Out of 130 molecules identified in serum and 199 molecules in urine, 7 molecules were selected and considered putative biomarkers after successive multivariate and univariate analyses. These were then selected for quantitative examination [22].

As shown in Table 1 presents the retention time (RT), the mass-to-charge ratio (*m*/*z* values), and the Area Under the Curve (AUC) values of the targeted metabolites.

Kynurenic acid, L-phenylalanine, and Met from serum demonstrated AUC values > 0.700.

Meanwhile, the urine Indoxyl sulfate, L-Phenylalanine, Arginine, and Kynurenic acid showed AUC values > 0.600.

The differentiation of the seven metabolites across groups (C, subgroup G1, G2, G3a, G3b, G4, and G5) was achieved through an untargeted univariate analysis, utilizing one-way ANOVA and Fisher’s LSD algorithms, as published before [22].

Based on the aforementioned data, the metabolites selected for targeted analysis included serum arginine, L-Methionine, L-Phenylalanine, Acetylcarnitine, kynurenic acid, and Indoxil Sulfate. Similarly, in urine, the metabolites comprised arginine, L-Methionine, L-Phenylalanine, Acetylcarnitine, kynurenic acid, and Indoxil Sulfate. The differentiation among subgroups, as determined by one-way ANOVA and Fisher’s LSD algorithms, and their disparities in MS peak intensities [22].

### 2.2. Quantitative Evaluation

#### 2.2.1. Calibrations and Validation Parameters

The delimitation of linear ranges, including calibration curves and equations with corresponding correlation coefficients (R^2^) values, along with the limit of detection (LOD) and limit of quantification (LOQ) for each standard, are presented in Table 2. The R^2^ exceeded 0.898 for all standards within their respective linear ranges, indicating a robust linear relationship. The LOD values were observed to fall within the range of 0.3–4 μM, while the LOQ values were within the range of 0.9–5.5 μM.

The liquid chromatography–mass spectrometry (LC-MS) method was validated for the quantitative assessment of metabolites through the controlled introduction of an internal standard (DOXO) and each of the pure standards to quality control (QC) extracts.

The recovery percentage, serving as an indicator of method reproducibility, was calculated, as presented in Table 3. An equal volume of QC extracts (0.3 mL) was supplemented with 0.2 mL from each of the eight standard solutions (50 µM Creatinine, 25 µM L-Phenylalanine, 12.5 µM L-Methionine, 5 µM Arginine, Dimethylarginine, Kynurenic Acid, L-Acetylcarnitine) and 3.4 µM of the internal standard DOXO. Table 3 illustrates the initial concentrations of metabolites post-mixing with the QC extract and the concentrations measured after the LC-MS analysis.

#### 2.2.2. Quantitative Evaluation of Serum and Urine Metabolites

The quantitative assessment of the seven metabolites derived from serum and urine was conducted based on the curve equation corresponding to each biomarker for each group (C, G1, G2, G3a, G3b, G4, and G5), as detailed in Table 4. The calibration curves are depicted in Supplementary Figure S1.

Additionally, a Mann–Whitney test was used to evaluate metabolite differences between subgroups; the data are presented in Table 5. Subsequently, the data obtained were correlated with references from the HMDB for each metabolite, as seen in Table 5.

## 3. Discussion

This study represents an advanced exploration into the intricate relationship be-tween kidney function and selective metabolite levels by conducting a comparative targeted metabolomic analysis of serum and urine samples from CKD patients as compared to healthy individuals. The objective was to validate the potential biomarkers initially identified through untargeted analysis using multivariate and univariate analysis [22] and further substantiate them through quantitative evaluation. The metabolites considered as potential biomarkers, including L-phenylalanine, L-methionine, Arginine, Indoxyl Sulfate, Kynurenic Acid, and L-acetylcarnitine, could potentially revolutionize CKD diagnosis and progression.

### 3.1. Free Amino Acids and Their Involvement in CKD

Our study identified a consistent decline in serum arginine concentrations (μM) when comparing the control group to the CKD subgroups. Moreover, statistical significance was observed between the control group and patients with early CKD stage (group G1). In contrast, arginine levels showed a slight decrease compared to controls and between CKD groups (G2 > G3a > G3b > G4 and G5 subgroups), though these changes were not statistically significant. A progressive increase in urinary arginine concentrations was observed across the CKD subgroups, from G1 to G5. Notable fluctuations in urinary arginine levels were also evident when comparing the control group with the G1 and G2 subgroups, respectively.

These findings, which align with previous studies reporting decreased serum arginine levels in CKD patients [23], support the validity of our research. A cross-reference of our results with the HMDB indicates that serum arginine levels typically range around 61.96 ± 18.03 µM, while urinary concentrations reach an average of 4.81 ± 2.45 µM/mmol creatinine [24].

Arginine, a semi-essential amino acid, is synthesized in humans through the intes-tinal–renal axis from precursors such as glutamine and glutamate. Its critical roles in the human body, particularly in protein synthesis and as a precursor for nitric oxide (NO) production by endothelial cells, have been well documented [25].

One of the most important functions of arginine is its involvement in NO synthesis via the enzyme nitric oxide synthase (NOS). In the kidney, multiple isoforms of NO play distinct roles. Endothelial-derived NO is essential for maintaining glomerular filtration rate, renal blood flow, and vascular tone. Neuronal NO, primarily localized in the macula densa, contributes to tubuloglomerular feedback regulation, thereby influencing glomerular hemodynamics. Inducible NO synthase (iNOS), produced in the glomerular mesangium, infiltrating macrophages, and tubular cells, is typically upregulated under pathological conditions, highlighting its role in renal injury and inflammation [25].

Several studies have demonstrated that impaired kidney function and conditions such as mesangial proliferative glomerulonephritis [26], lupus nephritis, and Wegener’s granulomatosis [27] are associated with increased production of iNOS. Additionally, in a model of anti-thymocyte serum (ATS)-induced glomerulonephritis, oral supplementation with 1% L-arginine during the induction phase increased glomerular NO synthesis, fibrosis, and proteinuria. Conversely, L-arginine supplementation after the injury induction phase was linked to reduced TGF-beta expression, decreased extracellular matrix accumulation, and attenuation of fibrosis [9].

At the tube level, arginine influences electrolyte management and tubular transport processes. It has been demonstrated to affect sodium reabsorption and ammonia genesis, processes crucial for acid–base equilibrium and homeostasis. Moreover, modifications in arginine metabolism may lead to heightened oxidative stress and inflammation, intensifying tubular injury and facilitating fibrosis. The dysregulation of the arginine-NO pathway is associated with increased oxidative stress, which exacerbates CKD progression by disrupting mitochondrial function and stimulating pro-inflammatory cytokine activation [28].

Collectively, these processes indicate that disruptions in arginine metabolism may exacerbate renal function decline in CKD, positioning it as a viable target for therapeutic intervention. Our investigation revealed evidence that substantiate its function in metabolic abnormalities associated with CKD.

A recent study also highlighted the significance of arginine deficiency in ADPKD. In this condition, the cell’s ability to synthesize arginine from intracellular precursors via urea-cycle enzymes is diminished, rendering these cells arginine dependent. ADPKD is further characterized by an accelerated metabolic rate, leading to increased arginine requirements [29].

Our study, correlated with previous research, suggests that arginine can be used as a potential biomarker for early CKD diagnosis. We hypothesize a bidirectional relationship between arginine and kidney function, where impaired kidney function reduces serum arginine levels. Conversely, oral arginine supplementation may exacerbate kidney function decline in specific contexts but offer protective benefits by mitigating further damage under certain conditions. This underscores the potential of arginine as a biomarker for early CKD diagnosis, though not for CKD progression.

Our study revealed elevated serum Met levels in patients with CKD compared to healthy controls, an increase evident even in the early stages (C > G1, G2). As CKD progressed, we noted that serum Met levels decreased progressively but without reaching statistical significance. In urine, Met concentrations progressively decreased from group C to group G5. These data align with previous studies associating higher Met levels with reduced eGFR [9,27,30]. Recent evidence also implicates methionine intake in the pathogenesis of CKD, potentially counteracting the benefits of a low-protein diet. Interestingly, urinary Met levels increased as CKD progressed, correlating with increased proteinuria.

Met is an essential amino acid precursor to cysteine, carnitine, creatinine, Hcy, and succinyl-CoA. Recent studies suggest that the Met contributes to oxidative stress by activating endogenous antioxidant enzymes and reducing oxidative damage [9]. An increase in reactive oxygen species (ROS) in the kidneys of the Met group has been observed, suggesting elevated oxidative stress, which can drive cellular damage and exacerbate CKD progression [31]. A study examining the impact of nickel sulfate on renal damage in rats revealed considerable glomerular and tubular degeneration and necrosis, marked by the death of tubular epithelial cells. The administration of Met exhibited a protective effect against such injuries, indicating its potential antioxidative characteristics in alleviating renal damage [32].

Moreover, methionine residues in proteins are prone to oxidation by reactive oxygen species, resulting in the formation of methionine sulfoxide. This alteration can modify protein function and has been associated with the regulation of vascular function and thrombosis. The reversible oxidation and reduction of methionine residues function as a mechanism for redox control in multiple biological processes, including those occurring in the kidneys [33].

This also regulates lipid metabolism, the innate immune system, and various metabolic processes. As a result, Met may influence the development and progression of chronic diseases, including CKD [34]. Dysfunctional autophagy is closely related to aging and age-related diseases, including kidney disease [35]. A study performed on mice showed that Met and its precursors activate the autophagy process from a low-protein diet [36]. Therefore, the accumulation of Met can be associated with CKD production due to stimulation of the autophagy process.

Furthermore, serum Met levels decrease as CKD advances while total homocysteine (tHcy) levels rise. Elevated tHcy is a well-known cardiovascular risk factor frequently associated with CKD. Reduced eGFR and proteinuria were linked to lower Met levels, suggesting a connection between methionine metabolism and renal function [37].

Our findings on Met metabolism and its role in CKD progression are significant. Disruptions in methionine metabolism have been linked to increased nicotinamide N-methyltransferase (NNMT) expression in the kidneys, a marker associated with renal fibrosis [38]. Met, a precursor of Hcy and cystathionine, plays a central role in this process [34]. The observed imbalance in the Met cycle and tHcy levels may reflect disturbances in the methylation cycle and broader metabolic dysregulation in CKD. These results underscore the importance of monitoring Met and tHcy levels in CKD management.

In our study, we observed a significant increase in plasma phenylalanine levels in patients with early CKD, which progressively increased as CKD advanced compared to control subjects. This suggests that phenylalanine metabolism may be disrupted in CKD patients. In contrast, the levels of phenylalanine in urine were significantly reduced in CKD patients, starting from the early stages of the disease, compared to the control group. These findings are in keeping with prior research [15,30].

L-phenylalanine is an indispensable amino acid vital for protein synthesis and serves as a precursor for several significant metabolites, such as tyrosine and neurotransmitters [39]. Current studies find that protein metabolism can be altered in CKD, leading to diminished clearance of amino acids and their metabolites. The current research demonstrates that plasma levels of L-phenylalanine diminish in the initial phases of CKD but rise as CKD progresses. This dynamic pattern underscores metabolic disturbances linked to deteriorating kidney function.

These findings align with prior research indicating that elevated L-phenylalanine levels in CKD patients contribute to oxidative stress and systemic inflammation, key factors exacerbating progression [30]. Furthermore, studies in human and animal models (e.g., rats) with renal failure have demonstrated that plasma and renal phenylalanine levels remain normal or slightly elevated. In contrast, tyrosine concentrations in plasma and skeletal muscle decrease, thus, leading to an increase in the phenylalanine-to-tyrosine ratio in plasma and muscle [40]. In CKD, the poor conversion of phenylalanine to tyrosine might result in altered plasma concentrations, potentially impacting both glomerular and tubular structures. The phenylalanine 4-hydroxylase enzyme converts dietary phenylalanine into tyrosine [14]. The interplay among phenylalanine, tyrosine, and renal impairment remains to be understood. Phenylalanine deficiency is correlated with increased oxidative stress.

The association between L-phenylalanine and tyrosine metabolism may indicate the influence of CKD on hydroxylase enzyme activity, which enables the transformation of phenylalanine into tyrosine. This imbalance may exacerbate metabolic and systemic problems associated with CKD [39,40]. Increased phenylalanine levels have been linked to heightened oxidative stress, a recognized factor in renal damage. Oxidative stress can cause damage to glomerular cells, compromising the integrity of the filtration barrier, and can also adversely affect tubular epithelial cells, leading to reduced reabsorption and secretion functions. Moreover, phenylalanine has demonstrated the ability to self-assemble into amyloid-like fibrils, which may accumulate in renal tissues and intensify structural damage [41].

These findings underscore the potential of L-phenylalanine as a biomarker for the diagnosis, monitoring, and potential risk stratification of CKD. Future studies should further investigate the processes connecting phenylalanine metabolism with oxidative and inflammatory pathways to understand its involvement in disease progression and therapeutic potential.

### 3.2. Uremic Toxins and Their Role in Early CKD Development

In the present study, KYNA levels were significantly elevated in patients with CKD as compared to healthy individuals. These levels were markedly increased even in the early stages of CKD compared to healthy controls. Across CKD stages, KYNA levels demonstrated a progressive rise parallel to the decline in kidney function. In urine samples, a slight increase in KYNA levels was observed in CKD patients compared to controls, following the pattern C < G1 < G2 < G3a < G3b < G4 < G5. These findings follow the results of previous research, supporting the correlation between worsening renal function and increased KYNA levels in serum and urine [16,42]. In contrast, Hirayama et al., in a study that included diabetic patients with high levels of albuminuria, found that serum levels of KYNA metabolites correlated negatively with GFR and positively with albuminuria [16].

Kynurenic acid is a metabolite in the kynurenine pathway, closely associated with tryptophan metabolism. Uremic encephalopathy is associated with the accumulation of many uremic toxins, including KYNA, which can disrupt neurotransmitter systems like glutamate transmission. This imbalance may result in cognitive and motor impairments frequently observed in CKD [43]. KYNA is an NMDA receptor antagonist, indicating its participation in neuroprotective and neurotoxic processes [44]. Elevated concentrations of KYNA in the blood and urine of CKD patients are likely due to reduced renal clearance and alterations in tryptophan metabolism, which amplify oxidative stress and inflammation [43]. Additionally, metabolites of the kynurenine pathway, including KYNA, are implicated in various biological processes linked to chronic diseases. These include impaired erythropoiesis, bone deterioration in CKD [45], atherosclerosis, carcinogenesis, and apoptosis [46]. Recent studies have emphasized its significance in numerous physiological and pathological processes, including renal function. As such, KYNA could serve as a diagnostic biomarker for CKD and a prospective therapeutic target. Moreover, elevated serum concentrations of IS is correlated with cardiovascular pathology, thereby being associated with adverse outcomes and heightened mortality rates in patients with CKD [21]. Numerous prior studies have reported increased serum and urinary levels of IS in patients with CKD [22].

Research on the direct effects of KYNA on glomerular and tubular structures is limited; nevertheless, several studies indicate a correlation between elevated urine levels of KYNA and negative renal outcomes. A study indicated that elevated urine levels of KYNA correlated with adverse renal and clinical outcomes in critically sick patients suffering from acute kidney damage, implying a possible involvement of KYNA in the advancement of kidney disease [47].

Specifically, KYNA contributes to increased oxidative stress in CKD, further aggravating the condition. Research by DiNatale et al. underscores that KYNA and other tryptophan metabolites act as endogenous activators of the aryl hydrocarbon receptor (AhR), which is involved in regulating immune responses, inflammation, and cellular homeostasis. This activation may be critical to CKD progression and its associated complications [48]. Furthermore, in an animal model study, increased activity of AhR was correlated with bone damage. Tryptophan metabolism and AhR were also correlated with kidney fibrosis [49].

These findings underline the importance of KYNA as a potential biomarker for CKD progression and suggest its involvement in the broader metabolic and inflammatory disturbances observed in CKD. Further studies are needed to elucidate its exact role and potential as a therapeutic target in managing CKD.

Our study highlights a progressive increase in serum IS concentrations across CKD stages, from group C to G1, G2, G3a, G3b, G4, and G5. A similar pattern was observed in urine, reflecting the gradual decline in renal clearance as CKD progresses. As shown in Table 4, IS has emerged as a promising biomarker in serum for monitoring CKD progression and severity. These findings align with previous research that reported elevated serum and urinary levels of IS in patients with CKD, emphasizing its accumulation due to a decrease in renal clearance and its potential role as a biomarker for disease progression [42,50]. Furthermore, Balint et al. reported elevated serum and urinary IS levels in DKD patients, starting as early as the normoalbuminuric stages [42]. In contrast, Niewczas et al. found that IS levels were not predictive of progression to ESRD in individuals with type 2 diabetes mellitus [51]. Interestingly, both human and animal studies have demonstrated that elevated levels of IS are significant predictors of all-cause mortality, underscoring its role as a critical uremic toxin associated with poor clinical outcomes [52].

Alterations in IS levels negatively impact the kidney’s glomerular and tubular structures. In the proximal tubules, IS intensifies oxidative stress by promoting excessive production of ROS [53]. This oxidative stress activates inflammatory pathways, including the nuclear factor-kappa B (NF-κB) pathway and the cAMP response element binding protein (CREB) in proximal tubular cells. As a result, pro-inflammatory cytokines such as interleukin-6 (IL-6) and tumor necrosis factor-alpha are released, further exacerbating inflammation and oxidative damage in the proximal tubules [53]. This cascade amplifies the oxidative burden in renal tissues, contributing to progressive kidney injury and dysfunction [54]. At the glomerular level, IS disrupts glomerular microvascularity by activating the AhR. This activation promotes endothelial dysfunction and contributes to a prothrombotic state through exacerbating vascular and renal damage [55].

Our study is in keeping with previous research and emphasizes the progressive increase in urinary levels of IS across the stages of CKD, from its early to the advanced phases. This accumulation results from declining renal clearance and is a promising biomarker for monitoring CKD progression and severity. Elevated levels of IS are not merely reflective of CKD presence but actively contribute to its progression through mechanisms such as oxidative stress, inflammation, and structural damage in both tubular and glomerular compartments of the kidney.

### 3.3. Carnitin’s Role in Early CKD Development and Progression

Our study revealed that serum concentrations of L-acetylcarnitine exhibited a slight increase during the early stages of CKD (subgroups G1 and G2) compared to healthy individuals. However, in more advanced stages (G3a, G3b, G4, and G5), these levels remained consistent with those observed earlier. In contrast, urinary levels of L-acetylcarnitine displayed a progressive increase from the G1 to the G5 group, suggesting a potential association with CKD progression. These findings are consistent with previous studies. Additionally, a cell culture experiment demonstrated that exogenous L-acetylcarnitine induced insulin resistance in skeletal muscle cells derived from patients with CKD, highlighting its potential metabolic effects in this population [56]. The role of acetylcarnitine in facilitating acetyl-CoA movement into mitochondrial matrices during fatty acid oxidation is well-established. However, elevated serum acetylcarnitine levels observed in individuals with CKD suggest a metabolic imbalance. This accumulation may inhibit carnitine acetyltransferase (CrAT) activity, disrupting skeletal muscle mitochondrial processes [57]. The resultant shift in CrAT activity in the reverse direction could contribute to mitochondrial dysfunction, exacerbating metabolic derangements in CKD patients [58]. These findings highlight the dual role of acetylcarnitine as both a metabolic facilitator and a potential contributor to mitochondrial dysregulation in the context of kidney disease [59]. Additionally, the data obtained from Liu et al. contradicts our findings, highlighting the differences in short- to medium-chain acylcarnitines, particularly tiglylcarnitine, between DKD and non-diabetic CKD cohorts [60]. L-acetylcarnitine has shown potential protective effects on glomerular and tubular structures. It helps maintain mitochondrial function and reduce oxidative stress, which are critical factors in preventing kidney damage [22].

Several studies have demonstrated that L-acetylcarnitine supplementation can improve mitochondrial respiration and energy production in proximal tubular cells, thereby reducing the risk of tubular injury and secondary glomerulosclerosis. Additionally, L-acetylcarnitine has been shown to mitigate oxidative damage and inflammation in renal tissues, which are common contributors to CKD progression [61].

These findings suggest that acylcarnitine may play a more specific role in DKD, potentially as a noninvasive biomarker to differentiate diabetic from non-diabetic forms of CKD. Further research is required to investigate this relationship and explore its clinical applicability in distinguishing between these two causes of CKD. This study has several limitations, including its cross-sectional design, small sample size, and the heterogeneity of the patient population.

The rationale for selecting these biomarkers stems from their proven diagnostic and prognostic value in CKD. By combining multiple biomarkers that target different stages of kidney injury, we aim to provide a comprehensive approach to disease assessment. This combinatory use not only enhances diagnostic accuracy but also improves the ability to predict disease progression and assess the effectiveness of interventions in specific CKD subgroups.

It should be underlined that Chen et al. identified serum 5-methoxytryptophan as a marker of CKD progression. While both studies focus on metabolites related to kidney function, Chen’s findings point to 5-methoxytryptophan as a more consistent and significant biomarker in serum [62]. By contrast, our study highlights arginine, Met, IS, and L-acetylcarnitine, particularly in urinary concentrations, as potential markers that show variability across different CKD stages. This difference in metabolite sources (serum vs. urine) and the statistical significance observed in their study vs. ours may reflect different underlying mechanisms in CKD progression, or they may indicate the need for further investigation into the clinical utility of both markers. While the seven markers in this study show promise as biomarkers, their accuracy and sensitivity in comparison to established markers such as neutrophil gelatinase-associated lipocalin, kidney injury molecule-1, and liver-type fatty acid binding protein, require further validation.

While the current study provides valuable insights into the role of specific metabolites, the clinical applicability of these findings will require validation across diverse patient populations and settings. A prospective approach would not only allow for the confirmation of these biomarkers’ predictive value but also this would facilitate a deeper understanding of their roles in disease progression, particularly in relation to early detection and therapeutic interventions in CKD. We aim to further explore this avenue by incorporating multi-center cohort studies, which will provide a broader and more robust dataset for validating these biomarkers in clinical practice.

Further validation in larger, multicenter, or prospective studies will be essential to confirm the clinical utility of these biomarkers and refine their role in personalized management of CKD.

## 4. Materials and Methods

### 4.1. Chemicals and Reagents

HPLC-grade formic acid was obtained from Sigma-Aldrich (St. Louis, MO, USA). HPLC/MS-grade formic acid and acetonitrile were provided by Fisher Scientific (Loughborough, UK). As an internal standard Doxorubicin hydrochloride (MW = 580) (injectable, 2 mg/mL Sun Pharmaceutical Industries, Goregaon, Mumbai, India) was used. The pure standards utilized for quantitative analysis were L-Methionine, from Amino acid standard H (product #20088, Thermo Scientific) (MW = 149 Da), L-Phenylalanine from Amino acid standard H (product #20088, Thermo Scientific) (MW = 165 Da), Acetyl-L-carnitine hydrochloride (J6153606; Alfa Aesar by Thermo Fisher) (MW = 203 Da), Kynurenic acid, >98.0% (HPLC) (H0303; TCI Chemicals, Portland, OR, USA) (MW = 189 Da), Arginine from Amino acid standard H (product #20088, Thermo Scientific) (MW = 174 Da), Creatinine > 98% product C4255, Sigma-Aldrich Chemie GmbH (MW = 113 Da), Indoxyl sulfate potassium salt, 97%, (A1707901; Alfa Aesar by Thermo Fisher), (MW = 213 Da). Other reagents: LC-MS grade MeOH, MeCN, and formic acid were purchased from Fisher Scientific (Loughborough, UK). Ultra-pure water was purified by a Milli-Q water system (Millipore, Milford, MA, USA). Instruments used in this study included a vortex mixer, Minicentrifuge Eppendorf (Thermo Fisher Scientific), UPLC-Q-TOF/MS (Bruker GmbH, Berlin, Germany).

### 4.2. Patients and Compliance with Ethical Standards

The study encompassed a cohort of 80 non-diabetic patients diagnosed with CKD, classified and staged in accordance with the KDIGO Guideline for the Diagnosis and Management of CKD [3] as described before [22]. Briefly, patients diagnosed with CKD (subgroups categorized into six groups such as the following: Group 1 (G1) included 15 patients with an eGFR of 90 mL/min/1.73 m^2^ or above; Group 2 (G2) comprised 15 patients with an eGFR ranging from 89 to 60 mL/min/1.73 m^2^; Group 3 (G3a) consisted of 17 patients with an eGFR between 59 and 45 mL/min/1.73 m^2^; Group 4 (G3b) contained 15 patients with an eGFR from 44 to 30 mL/min/1.73 m^2^; Group 5 (G4) incorporated 15 patients with an eGFR from 29 to 15 mL/min/1.73 m^2^; and Group 6 (G5) included 14 patients with an eGFR less than 15 mL/min/1.73 m^2^) were recruited, and the health control (HC) groups were age and gender matched, without evidence of risk factor. The demographic, biological, and clinical data for CKD groups (G1 to G5) and C group are presented in Table 6.

The Research Ethics Committees from Timişoara University Hospital (Approval No. 222/04.02.2021) and the Ethics Committee for Scientific Research at “Victor Babes” at the University of Medicine and Pharmacy Timisoara (Approval No. 54/09.11.2020) approved this study. All participants signed an informed consent document. All procedures were conducted according to the criteria set by the Declaration of Helsinki.

The exclusion criteria were the presence of diabetic kidney disease, neoplasia, acute kidney injury, and acute kidney disease, and the need for renal replacement therapies. Blood and urine samples were collected in the morning following a 12 h fast. Concurrently, other clinical and biological data were gathered, recorded, and presented in Table 6 for both groups. Our analysis showed that there were no statistically significant demographic differences (e.g., age, sex, comorbidities) among the CKD subgroups that could have influenced the observed metabolic changes. The statistical analysis was performed using a one-way ANOVA with Bonferroni correction, a Chi-squared test, and a Kruskal–Wallis test, which allowed for the initiation of clinical-biological features between subgroups, as presented in Table 6.

### 4.3. Sample Collection and Processing

The blood serum was collected by venipuncture in sterile vacutainers without an anticoagulant, and the serum was kept at −80 °C until analysis. They were labeled using confidential numerical codes. The urine samples were collected in the morning in sterile vials. A volume of 0.8 mL mix of pure HPLC-grade Methanol and Acetonitrile (2:1 *v*/*v*) was added for each volume of 0.2 mL of serum and 0.2 mL urine, respectively. In each case, the mixture was vortexed to precipitate proteins, ultrasonicated for 5 min and kept at −20 °C for 24 h to increase the protein precipitation. The supernatant was collected after the centrifugation at 12,500 rpm for 10 min (4 °C) and filtered through Nylon filters (0.2 μm). Finally, it was placed in glass micro vials and introduced in the autosampler of ultra-high-performance liquid chromatography (UHPLC) before injection. The supernatant was transferred to an autosampler vial for HPLC-MS analysis. QC samples from a mix of 0.1 mL from each serum or urine sample were also obtained and used as representative generic samples, which were injected at the beginning and end and as every 10th injection while analyzing the study samples.

### 4.4. UHPLC-QTOF-ESI ± MS Analysis

The metabolomic profiling was performed by ultra-high performance liquid chromatography coupled with electrospray ionization quadrupole time-of-flight mass spectrometry (UHPLC-QTOF-ESI ± MS) using a Thermo Fisher Scientific UHPLC Ultimate 3000 instrument equipped with a quaternary pump, Dionex delivery system, and MS detection equipment with MaXis Impact (Bruker Daltonics, Berlin, Germany). The metabolites were separated on an Acclaim C18 column (5 μm, 2.1 × 100 mm, pore size of 30 nm) (Thermo Scientific) at 28 °C. The mobile phase consisted of 0.1% formic acid in water (A) and 0.1% formic acid in acetonitrile (B). The elution time was set for 20 min. The flow rate was set at 0.3 mL·min^−1^ for serum samples and 0.8 mL·min^−1^ for urine samples. The gradient for serum samples was as follows: 90 to 85% A (0–3 min), 85–50% A (3–6 min), 50–30% (6–8 min), 30–5% (8–12 min), and afterward increased to 90% at min 20. The gradient for urine samples was as follows: 90 to 85% A (0–3 min), 85–30% A (3–6 min), 30–10% (6–8 min), isocratic until min 12, and then increased until 90% at min 20. The volume of injected extract was 5 µL, and the column temperature was 25 °C. Two categories of QC samples were obtained by mixing similar volumes of serum and urine, respectively, to calibrate the separations. Doxorubicin hydrochloride (*m*/*z* = 581.3209) (2 mg/mL) was added in parallel to QC samples as an internal standard (IS).

The applied MS parameters were ionization mode positive (ESI+), MS calibration with Natrium formate, capillary voltage 3500 V, pressure for the nebulizing gas 2.8 Barr, drying gas flow 12 L/min, and drying temperature 300 °C. The *m*/*z* values to be separated were set between 60 and 600 Daltons. The control of the instrument and the data processing was performed using the specific software TofControl 3.2, Chromeleon, and HyStar Data Analysis 4.2, respectively (Bruker, Daltonics, Berlin, Germany).

### 4.5. Data Processing and Statistical Analysis

In the subsequent phase, untargeted analysis was performed for the same groups and were previously described [22]. For the statistical analysis of targeted metabolites, the matrices included for the above-mentioned metabolites were selected (*m*/*z* values vs. MS peak intensity, as .csv file) and applied the Metaboanalyst 5.0 platform for multivariate and univariate analysis (https://www.metaboanalyst.ca, 7 January 2025).

Differences between the two groups (controls versus subgroups G1, G2, G3a, G3b, G4, and G5) were first analyzed using the multivariate analysis by fold change, PCA, and PLSDA score plots, including VIP values. Volcano plots were generated with the log2 fold change values and Bonferroni-adjusted *p*-values. The value of *p* < 0.05 was defined as statistically significant.

To test each metabolite’s discriminatory capacity, we performed receiver operating characteristic (ROC) analysis. For AUC values higher than 0.8, the metabolite was considered to have a very high prediction effect on disease and can be used as a candidate biomarker for further study.

In the second step, the one-way ANOVA univariate analysis aimed to discriminate between the controls and the subgroups of patients (G1, G2, G3, etc.). The PCA and PLSDA score plots including VIP values, cross-validation parameters, as well as the mean decrease accuracy (MDA) scores by Random Forest analysis were performed.

According to the data collected from untargeted metabolomics, there was a targeted number of six specific molecules in plasma, and their MS peak intensities were considered to be compared in the different groups of patients, e.g., between the healthy subjects group C and the pathological ones. The mean values of PI and their SD were calculated for these specific biomarkers selected by the untargeted metabolomics and in agreement with recent literature data. For quantitative analysis, the calibration curves were built with pure standards.

### 4.6. Metabolites Identification

According to the data obtained from the untargeted metabolic fingerprints of samples, there were selected metabolites that showed statistical significance as putative biomarkers of differentiation between groups. Therefore, we selected the matrices, which were included for these metabolites, for the statistical analysis using the Metaboana-lyst 5.0 platform for multivariate and univariate analysis (https://www.metaboanalyst.ca, 7 January 2025).

The chemical information about metabolites was searched through the human metabolome database HMDB (http://www.hmdb.ca, 7 January 2025), PubChem (https://pubchem.ncbi.nlm.nih.gov), and Lipidmaps (https://www.lipidmaps.org), considering a deviation of the *m*/*z* value of 0.05. The identification results are proved by combining the exact number (*m*/*z*) with the ionization method and comparing the primary and secondary mass spectra information of the differential metabolites with the theoretical fragments of the HMDB search results.

According to the data collected from untargeted metabolomics, a number of six specific molecules in serum were targeted, and their MS peak intensities were considered to be compared in the different groups of patients, e.g., between the groups C and P. The mean values of PI and their standard deviations SD were calculated for these specific biomarkers selected by the untargeted metabolomics and in agreement with recent literature data. For quantitative analysis, the calibration curves were built with pure standards.

The chemical information of differential metabolites was searched through the human metabolome database (HMDB; http://www.hmdb.ca/, 7 January 2025) and Lipidmaps (https://www.lipidmaps.org/). The input was the precise molecular mass, and the ionization method considered that the deviation of the *m*/*z* value does not exceed 0.05 Da. The identification results are proved by combining the exact number of charges and the ionization method that meets the experimental conditions.

Moreover, to explore how the major metabolic pathways related to the differential metabolites were affected, metabolic pathway analysis was performed by the MetaboAnalyst 5.0 71 platform (http://www.metaboanalyst.ca, 7 January 2025).

### 4.7. Quantitative Evaluation

Preparation of calibration solution and QC samples. For calibration, stock solutions of pure standards were first obtained: L-Methionine 1 mM, L-L-Phenylalanine 1 mM, Kynurenic acid 1 mM, Arginine 1 mM, Dimethylarginine 1 mM, Creatinine 2mM, L-Acetylcarnitine 1 mM, and Kynurenic acid 1 mM dissolved in ultrapure water. The stock solutions were successively diluted in the mix of methanol–acetonitrile 2:1 to obtain the series of working solutions at different concentration levels for external calibration. In parallel, volumes of 0.3 mL QC deprotonated samples were spiked with different volumes of standard solutions.

The method validation was achieved according to the “Guidance for Industry Bioanalytical Method Validation” recommended by the US Food and Drug Administration, named the UHPLC-QTOF-ESI ± MS method. The linearity, specificity, precision, accuracy, LOD, and LOQ were checked and recorded. Two calibration curves were generated: (1) by an external standard calibration curve, made by diluting standard solutions in the mobile phase and (2) by an internal standard curve, whose linearity was determined for QC samples spiked with different volumes of standard solutions. The mean peak area of three replicate measurements at each concentration was calculated.

The LOD was considered as the lowest concentration of analyte in the test sample that can be reliably distinguished from zero to signal/noise ratio ≥ 10. The LOQ was the lowest concentration of analyte that can be determined with an acceptable repeatability and trueness (signal/noise ratio ≥ 10 and SD values ≤ 40%).

## 5. Conclusions

In conclusion, our investigation highlights that several serum and urine metabolites, including L-phenylalanine, L-methionine, arginine, indoxyl sulfate, kynurenic acid, and L-acetylcarnitine, may serve as potential biomarkers in the pathogenesis and diagnosis of early CKD. Moreover, urine research indicated that IS could serve as a potential biomarker for the early detection of CKD. The quantification of serum and urinary metabolites in patients with CKD provided new insights into the involvement of various metabolic pathways in CKD development, potentially facilitating their standardization and application in individualized metabolic biological panels for early CKD diagnosis.

## Figures and Tables

**Table 1:** The classification of the seven metabolites selected from serum and urine discriminates the group CKD from group C. The *m*/*z* values, RT, and AUC values for these molecules in serum and urine are presented.

*m*/*z*	Identification Serum	RT (min)	AUC Serum	AUC Urine
114.0991	Creatinine	0.7	0.591	0.509
150.1231	L-Methionine	1.7	0.732	0.507
166.0979	L-Phenylalanine	1.3	0.806	0.660
175.1305	Arginine	1.0	0.502	0.658
190.0629	Kynurenic acid	11.4	0.820	0.619
214.2550	Indoxyl sulfate	11.6	0.631	0.790
204.1367	Acetylcarnitine	1.2	0.592	0.573

Legend: *m*/*z*—the mass-to-charge ratio; RT—retention time; AUC—the Area Under the Curve.

**Table 2:** Validation parameters represented by linear range (µM), curve equation, R^2^, LOD-LOD (µM), and LOQ (µM), for each of the molecules selected as potential biomarkers.

Name	Linear Range	Curve Equation	R^2^	LOD	LOQ
Creatinine	5–25	y = 2693.3x − 254.5	0.994	0.5	1.0
L-Methionine	2.5–20	y = 3260x + 40	0.998	0.5	1.0
L-Phenylalanine	5–25	y = 12,165x + 4223.1	0.995	0.5	1.0
Arginine	2–40	y = 2476.6x + 427.61	0.999	0.2	0.8
Kynurenic acid	0.1–2	y = 25,274x − 2.461	0.999	0.08	0.1
Indoxyl sulfate	0.5–25	y = 8157x − 1240.8	0.999	0.2	0.8
L-Acetylcarnitine	1–5	y = 36,813x − 3879.2	0.994	0.2	0.8

Legend: LOD—Limit of Detection, LOQ—Limit of Quantification, R^2^—correlation coefficients.

**Table 3:** The recovery percentage (%) was determined based on the measured concentrations of the internal standard (IS) and each metabolite (pure standard) in comparison to their initial concentrations following their addition to the QC extract.

Metabolite	Initial Concentration (μM)	Measured Concentration (μM)	Recovery (%)
Creatinine	20	17.5	87.5
L-Methionine	5	4.55	91.0
L-Phenylalanine	10	8.55	85.5
Arginine	2	1.85	92.5
Kynurenic acid	2	1.65	82.5
Indoxyl sulfate	5	4.8	96.0
L-Acetylcarnitine	2	1.85	92.5
IS	1.4	1.25	89.3

Legend: IS: indoxyl sulfate.

**Table 4:** The mean values and standard deviations (±SD) of serum and urine concentrations (mM/mM creatinine) of the potential biomarkers targeted in this study for groups C and subgroups G1, G2, G3a, G3b, G4, and G5.

BLOOD (mM)	Control	G1	G2	G3a	G3b	G4	G5
L-Methionine	36.73 (7.5) ^♦,^*	74.07 (5.3) ^▲,♣,♦,‡,✧^	62.06 (17.6) ^♣,♦,✧^	34.83 (18.33) ^♣,†^	35.92 (19.2) ^♣,†^	59.84 (13.4)	49.30 (16.96)
L-Phenylalanine	77.21 (24.7) ^†,⁑^	104.80 (49.4)	110.17 (30.9)	107.61 (24.53)	105.74 (29.95)	119.90 (24.6)	105.76 (48.72)
Arginine	50.03 (10)	49.59 (17.5)	59.54 (20.8)	47.63 (17.20)	36.18 (14.33)	61.96 (18)	53.75 (20.66)
Kynurenic acid	5.27 (1.3) ^♦^	12.83 (3.5) ^▲,♣,♦,○,✧^	9.29 (2.67) ^▲,†,✧^	6.57 (1.55)	6.24 (1.40)	8.71 (2.73)	6.46 (1.93)
Indoxyl sulfate	4.83 (1.4) ^†,^*	6.51 (2.3) ^♣,†,○,✧^	6.41 (1.60) ^♣,♦,○,✧^	3.94 (1.84) ^♣,♦^	3.84 (1.95) ^♣,♦^	6.11 (0.47) ^▲^	5.80 (1.23)
L-Acetylcarnitine	1.80 (0.2)^♦,⁑^	4.24 (0.2) ^♣,♦,✧^	4.30 (0.12) ^♣,♦^	1.77 (0.06) ^♣,♦^	1.83 (0.10) ^♣,♦^	4.18 (0.34)	3.75 (1.47)
URINE (μM/mM creatinine)							
L-Methionine	5.99 (5.18) ^▲^	6.42 (2.51) ^▲^	4.83 (2.61) ^○^	5.07 (2.37)	7.72 (6.24)	6.77 (2.89)	8.74 (5.94)
L-Phenylalanine	5.69 (5.08) ^†,○^	2.18 (1.79) ^♣^	4.17 (5.56) ^▲^	7.86 (4.74) ^○^	2.95 (2.87)	2.52 (1.99) ^▲^	6.45 (5.70)
Arginine	4.09 (1.69) ^†^	2.83 (0.84) ^▲^	3.90 (1.34)	3.47 (2.33)	4.81 (2.45)	4.64 (3.04)	3.74 (5.39)
Kynurenic acid	0.33 (0.19) ^▲^	0.40 (0.18) ^†^	0.47(0.19) ^†^	0.41 (0.20) ^○^	0.82 (0.64)	0.52 (0.43)	0.62 (0.41)
Indoxyl sulfate	0.72 (0.43) ^†,♣,♦,‡,✧^	1.12 (0.25) ^▲,♦,✧^	1.22 (0.43) ^▲,†^	1.87 (1.02)	2.82 (1.88)	1.75 (0.73)	2.51 (1.61)
L-Acetylcarnitine	0.14 (0.06) ^†^	0.14 (0.04) ^†^	0.15 (0.04) ^†^	0.14 (0.07) ^○^	0.25 (0.15)	0.17 (0.08)	0.20 (0.14)

Legend: Statistical significance between healthy controls and G1 group, ♦ *p* < 0.001; † *p* > 0.001 and *p* < 0.05; statistical significance between healthy controls and G2 group, ▲ *p* > 0.001 and *p* < 0.05; statistical significance between healthy controls and G3a group, ♣ *p* < 0.001; statistical significance between healthy controls and G3b group, ♦ *p* < 0.001; † *p* > 0.001 and *p* < 0.05; statistical significance between healthy controls and G4 group, ‡ *p* < 0.001; ○ *p* > 0.001 and *p* < 0.05; statistical significance between healthy controls and G5 group: ✧ *p* < 0.001; statistical significance between G1 group and G2 group, ▲ *p* > 0.001 and *p* < 0.05; statistical significance between G1 group and G3a group, ♣ *p* < 0.001; statistical significance between G1 and G3b group, ♦ *p* < 0.001; † *p* > 0.001 and *p* < 0.05; statistical significance between G1 and G4 group and macroalbuminuric group, ‡ *p* < 0.001; ○ *p* > 0.001 and *p* < 0.05; statistical significance between G1 and G5 group, ✧ *p* < 0.001; statistical significance between G2 group and G3a group, ♣ *p* < 0.001, ▲ *p* > 0.001 and *p* < 0.05; statistical significance between G2 and G3b group, ♦ *p* < 0.001; † *p* > 0.001 and *p* < 0.05; statistical significance between G2 and G4 group and G5 group, ○ *p* > 0.001 and *p* < 0.05; statistical significance between G2 and G5 group, ✧ *p* < 0.001; G3a group and G3b group, ▲ *p* > 0.001 and *p* < 0.05; statistical significance between G3a group and G4 group, and G3a-G5 group, ♣ *p* < 0.001; statistical significance between G3a and G5 group, ♦ *p* < 0.001; † *p* > 0.001 and *p* < 0.05; Statistical significance between G4 group and G5 group, ▲ *p* > 0.001 and *p* < 0.05; statistical significance between healthy controls vs. G1 group vs. G2 group vs. G3a group; G1 group vs. G2 group vs. G3a group vs. G3b group vs. G4 group vs. G5 group; * *p* < 0.001, ⁑ *p* > 0.001 and *p* < 0.05; *p*-values based on Mann–Whitney test.

**Table 5:** The normal range for targeted metabolites is based on the HMDB database.

Biomarker	Normal Range Based on HMDB		References
	Serum (μM)	Urine (μM/μM Creatinine)	
L-Methionine	25–35	1–4	https://hmdb.ca/metabolites/HMDB0000696, 7 January 2025
L-Phenylalanine	40–75	3–11	https://hmdb.ca/metabolites/HMDB0000159, 7 January 2025
Arginine			
Kynurenic acid	0.03 ± 0.007	1–1.6	https://hmdb.ca/metabolites/HMDB0000715, 7 January 2025
Indoxyl sulfate			
L-Acetylcarnitine	1–4	10–20	https://hmdb.ca/metabolites/HMDB0000682, 7 January 2025

**Table 6:** The demographic, biological, and clinical data for CKD groups (G1 to G5) and C group.

	C	CKD					
		G1	G2	G3a	G3b	G4	G5
Participants	20	12	15	17	15	15	14
Sex (M)	12	7	9	6	7	8	6
Age (y)	55.85 ± 7.25	39.92 ± 10.8	53.6 ± 15.4	55.1 ± 15.2	58.9 ± 14.4	61.2 ± 14.8	63.6 ± 12.6
BMI (kg/m^2^)	25.35 ± 8.5	26.42 ± 3.1	26.9 ± 1.7	27.9 ± 1.9	28.5 ± 2.3	27.3 ± 3.2	28.6 ± 2.2
Comorbidities							
Glomerulonephritis	0	3	5	1	0	5	3
Hypertension	0	12	15	17	15	15	14
Acquired Solitary Kidney	0	0	0	0	0	2	1
Serum creatine (mg/dL)	0.73 ± 0.08	1.46 ± 2.1	1.4 ± 0.3	1.6 ± 0.7	1.7 ± 0.3	6.5 ± 13	5.19 ± 0.8
e-GFR(mL/min/1.73 s)	97.93 ± 11.71	101.9 ± 12.2	65.1 ± 12.9	49 ± 10.6	39.9 ± 5	21.3 ± 11.6	12.9 ± 11.8
uACR	14.67 ± 6.4	449.7 ± 1177.3	1252.3 ± 1625.7	630.6 ± 709.2	672.9 ± 1509.1	747.9 ± 884	1102.9 ± 1365.6

Legend: M: male; BMI: body mass index; e-GFR: estimated glomerular filtration rate; uACR: urine albumin–creatinine ratio.

## Data Availability

The original contributions presented in this study are included in the article and Supplementary Materials. Further inquiries can be directed to the corresponding author.

## Supplementary Materials

The following supporting information can be downloaded at: https://www.mdpi.com/article/10.3390/ijms26072862/s1.

## Abbreviations

The following abbreviations are used in this manuscript:

CKD	chronic kidney disease
eGFR	estimated filtration rate
LOD	limit of detection
LOQ	limit of quantification
ESRD	end-stage renal disease
ADPKD	autosomal dominant polycystic kidney disease
DR	diabetic retinopathy
Met	L-methionine
Hcy	homocysteine
IS	indoxyl sulfate
KYNA	kynurenic acid
M	male
BMI	body mass index
uACR	urine albumin–creatinine ratio
UHPLC	ultra-high performance liquid chromatograph
UHPLC-QTOF-ESI ± MS	liquid chromatography system coupled with electrospray ionization quadrupole time-of-flight mass spectrometry
QC	quality control
*m*/*z*	mass-to-charge ratio
ROC	receiver Operating Characteristic
AUC	area under the curve
MS	mass spectrometry
PI	peak intensities
RT	retention time
HMDB	human metabolome database
