# Supplementary material for: Targeted Analysis of Serum and Urinary Metabolites for Early Chronic Kidney Disease
Source: Int J Mol Sci. 2025 Mar 21;26(7):2862. doi: 10.3390/ijms26072862 (PMC11989156; doi:10.3390/ijms26072862)
Supplement: Supplementary file 1 [file ijms-26-02862-s001.zip › ijms-3509700-supplementary.pdf]

Figure S1. Graphic representation of the differences (expressed in MS peak intensities) between the groups C, G1, G2, G3a, G3b, G4 and G5, for each of the eight urine potential biomarkers. The original and normalized (sample normalization by median values) are presented

Calibration curves for the targeted metabolites

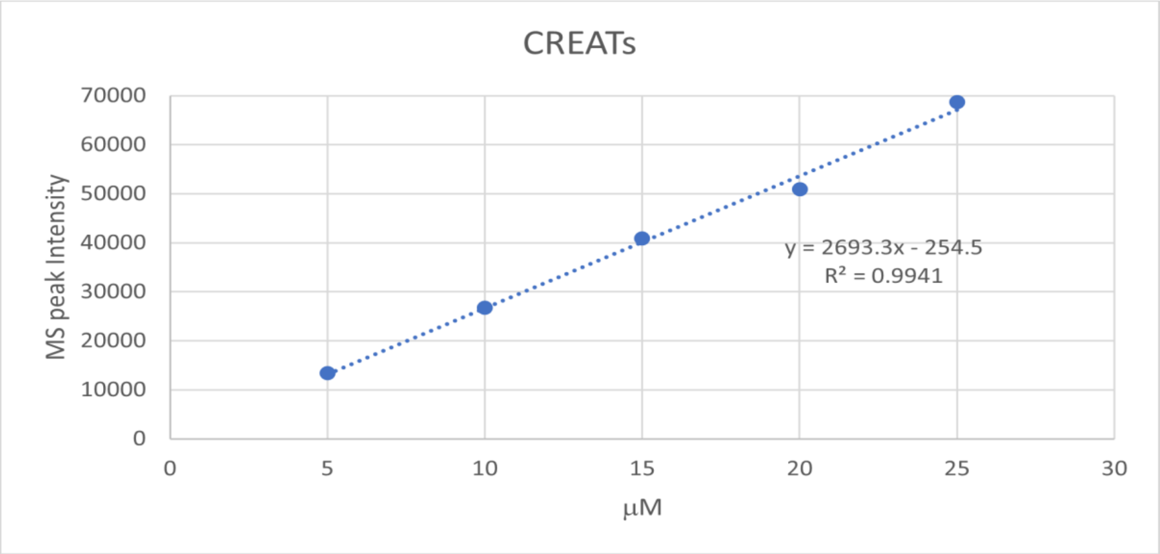

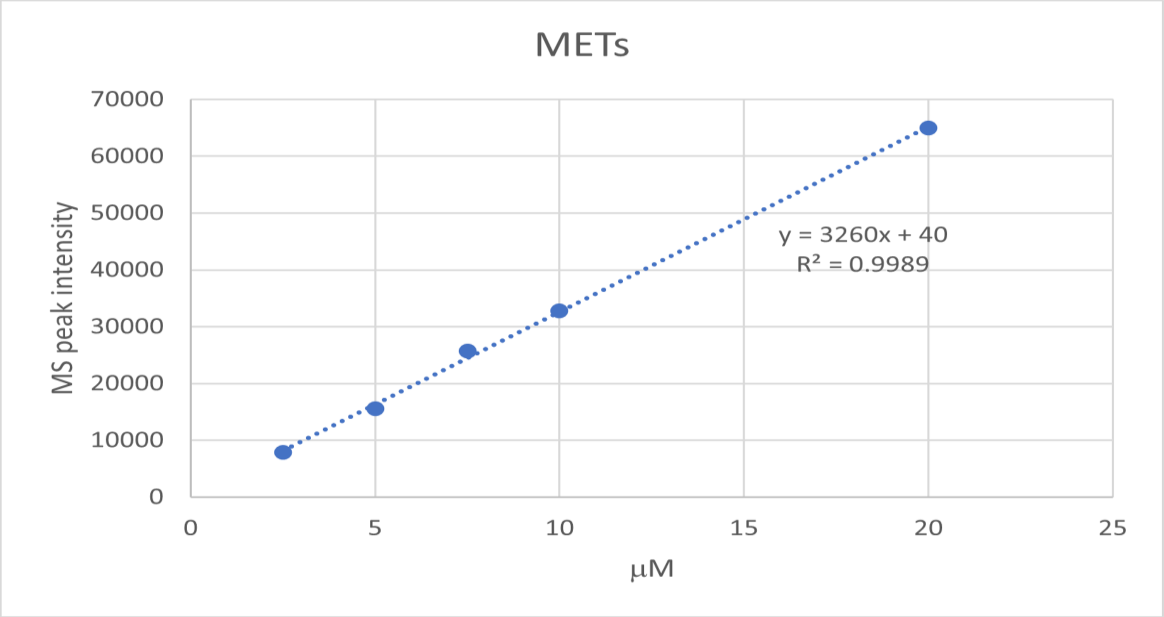

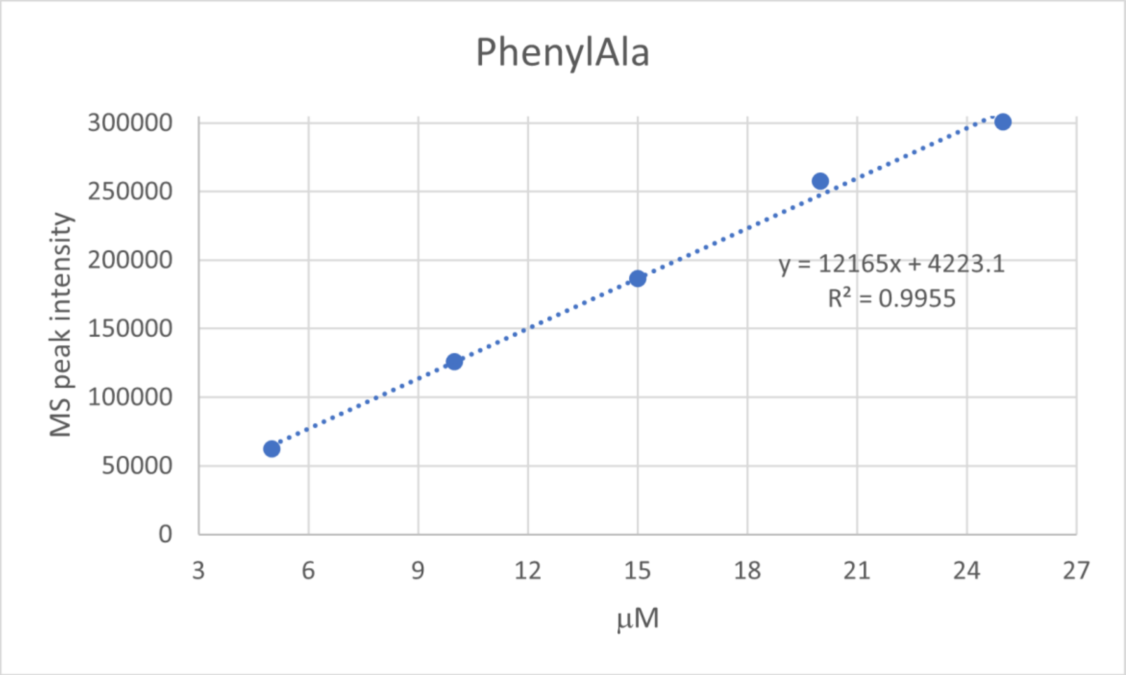
PhenylAla
300000
250000
200000
150000
100000
50000
0
MS peak intensity
y = 12165x + 4223.1
R² = 0.9955
3
6
9
12
15
18
21
24
27
µM

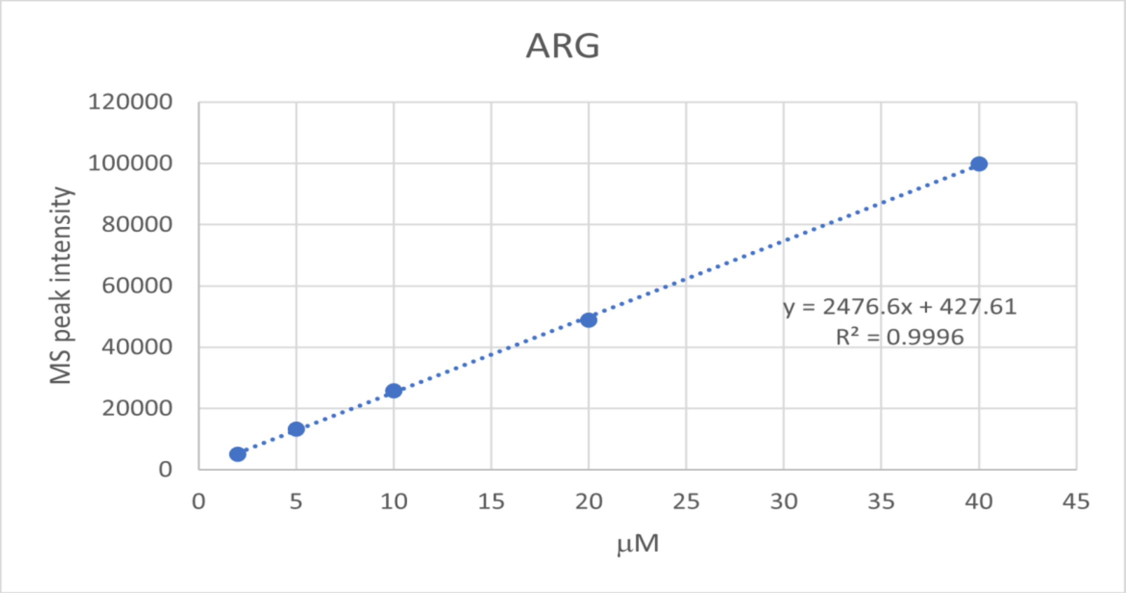
ARG
120000
100000
80000
60000
40000
20000
0
MS peak intensity
y = 2476.6x + 427.61
R² = 0.9996
0
5
10
15
20
25
30
35
40
45
µM

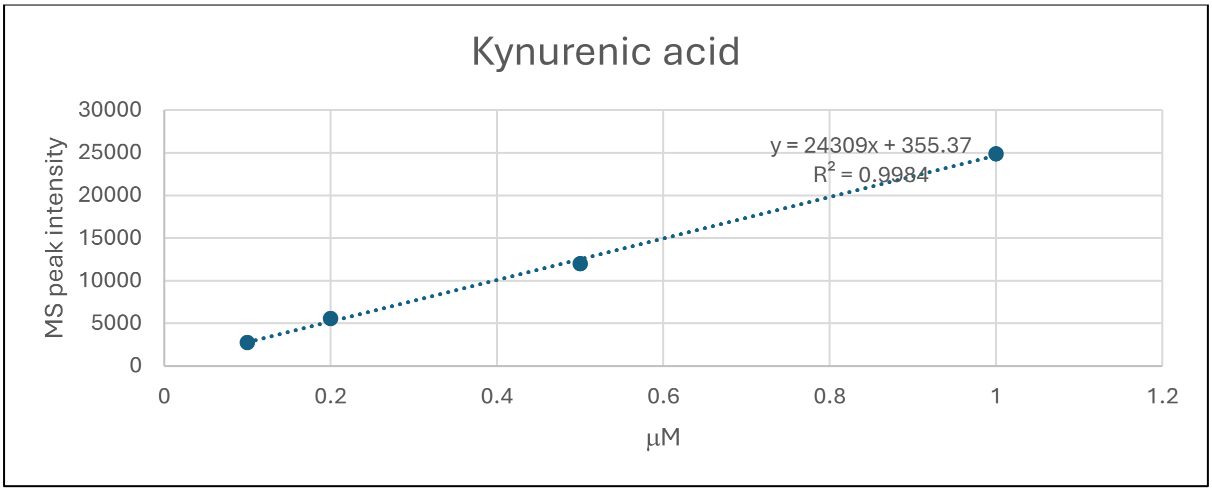
Kynurenic acid
MS peak intensity
30000
25000
20000
15000
10000
5000
0
0
0.2
0.4
0.6
0.8
1
1.2
µM
y = 24309x + 355.37
R² = 0.9984

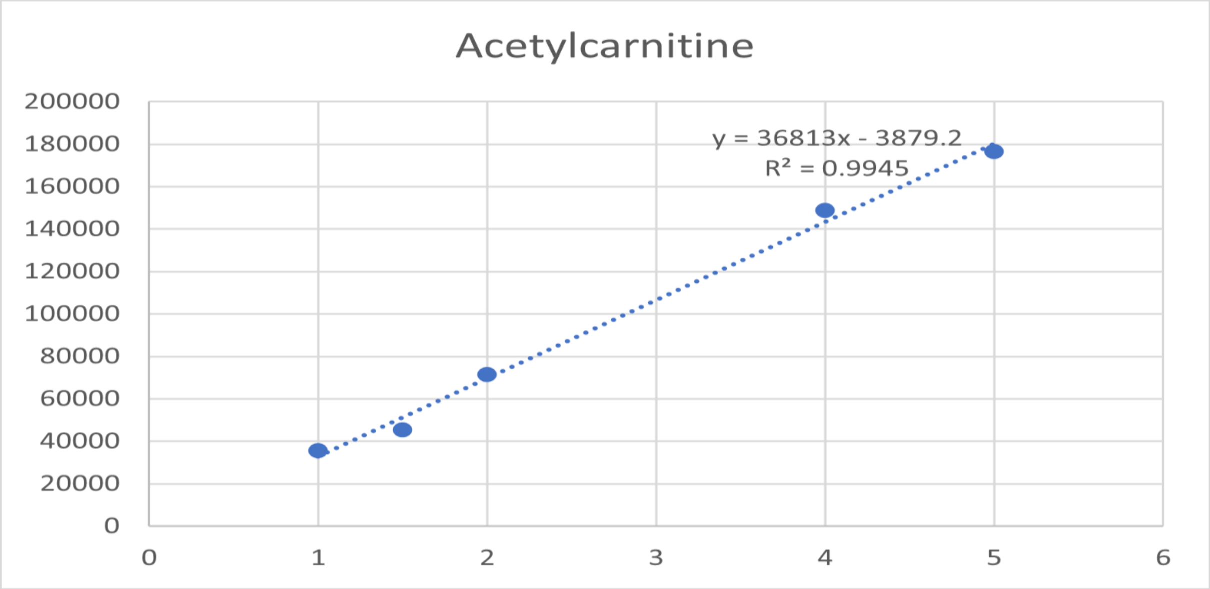
Acetylcarnitine
200000
180000
160000
140000
120000
100000
80000
60000
40000
20000
0
0
1
2
3
4
5
6
y = 36813x - 3879.2
R² = 0.9945

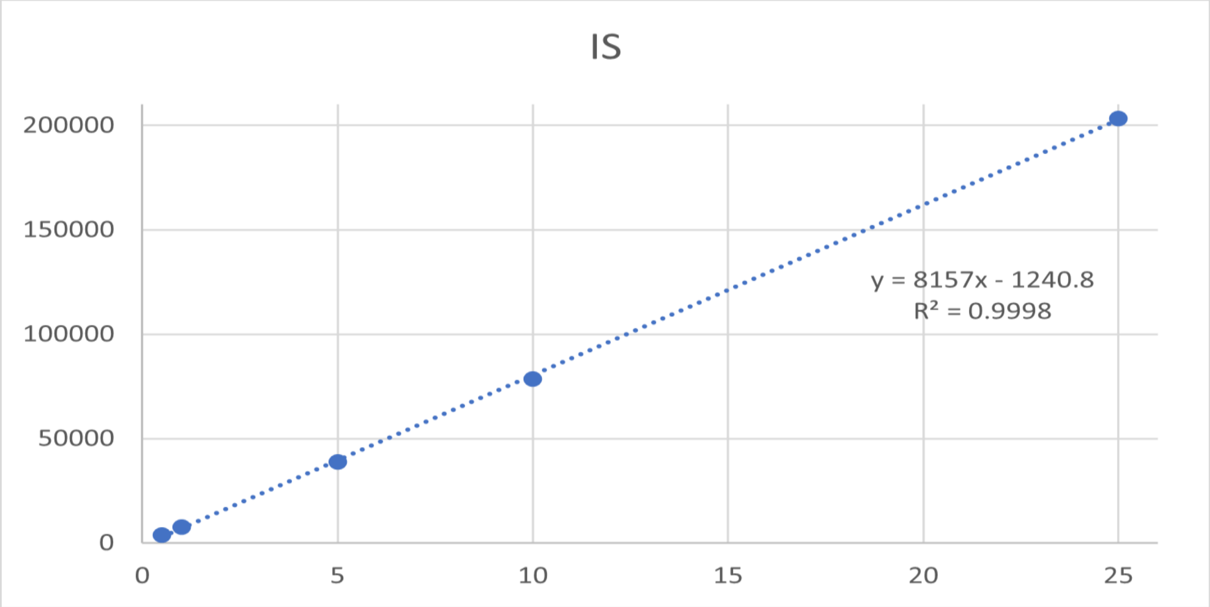
IS
200000
150000
100000
50000
0
0
5
10
15
20
25
y = 8157x - 1240.8
R² = 0.9998
